# The Effects of Workplace Loneliness on the Psychological Detachment and Emotional Exhaustion of Hotel Employees

**DOI:** 10.3390/ijerph19095228

**Published:** 2022-04-25

**Authors:** Yoon-Sik Jung, Hyo-Sun Jung, Hye-Hyun Yoon

**Affiliations:** 1 Department of Culinary Arts and Food Service Management, Kyung Hee University, Seoul 02447, Korea; coolx2ee@naver.com; 2 Center for Converging Humanities, Kyung Hee University, Seoul 02447, Korea; chefcook@khu.ac.kr

**Keywords:** workplace loneliness, psychological detachment, emotional exhaustion, hotel industry

## Abstract

This study was aimed at establishing whether loneliness among hotel employees in the workplace affects their psychological and emotional experiences by empirically investigating their perceptions of negative situations. A self-administered questionnaire was distributed to 300 hotel employees, after which confirmatory factor analysis was conducted to reassess the reliability and validity of the measured questionnaire items. A model of workplace loneliness, psychological detachment, and emotional exhaustion was developed and examined through structural equation modeling. The results showed that the hotel employees experienced workplace loneliness and expressed a desire to be psychologically detached from their jobs for recovery. Workplace loneliness also contributed to emotional exhaustion. Theoretical and practical implications, as well as limitations and future research directions, are discussed.

## 1. Introduction

With the advent of globalization, considerable changes in communities and rapid economic advancement have brought forth many employee-related issues in the workplace [1]. Work occupies a considerable part of an individual’s lifetime, rendering social relationships in workplaces increasingly important for both professional and individual lives [2]. In such environments, experiencing loneliness reveals that people are in some way dissatisfied with their relationships and indicates a desire for expanded social connections [3,4]. On this basis, workplace loneliness is a disagreeable emotion that stems from discontent with existing social relationships [5] or a dearth in such connections [6] in the workplace. In the hospitality industry, hotel employees meet other people (customers, fellow workers, supervisors) every day, and this engagement is the most important part of their work. Work encompasses us within a social boundary, and there are few, if any, businesses where one accomplishes responsibilities completely independently [7]. Considering the frequency of contact between hotel employees and other individuals, complaints about social relationships often occur. Workplace loneliness has serious effects on organizations. It reduces creativity [8], erodes performance [3,9], and enhances turnover intention [10]. The sense of isolation arising from loneliness diminishes solidarity between members of an organization [11]. Employees who experience high degrees of loneliness also grapple with considerable emotional exhaustion [12,13]. This complication is why it is essential to explore workplace loneliness, as the insights derived can help organizations maintain engagement among employees and enable these individuals to cultivate positive relationships.

Employees who want to recover from negative situations at work can do so by recharging. Employees who endure unfavorable circumstances, such as emotional problems and heavy workloads, often require substantial recovery time [14,15] because they need to expend extra effort and regulate their emotions to perform their duties [16]. An especially important requirement is for employees to psychologically disconnect themselves from their jobs during nonwork hours [17]. Sonnentag [18] offered that recovery experiences were positively connected to ensuing on-the-job behavior. As reported by Etzion et al. [19] and Sonnentag and Bayer [20], psychological detachment from the workplace during off hours is extremely significant for recovery to occur. In the same vein, Kilroy et al. [21] and Muhamad et al. [22] asserted that high levels of psychological detachment increase the negative relationship between burnout and emotional exhaustion. As can be seen, psychological detachment is an important aspect through which employees can minimize negative experiences and replenish their resources. Adding to these insights, Sonnentag [18] indicated that recovery experiences are positively connected to ensuing on-the-job behaviors. Although interest in psychological problems has grown, few empirical studies have been directed toward workplace loneliness and the processes by which this condition occurs. Research has also rarely associated emotional problems in the workplace with co-worker relationships. To address these deficiencies, the present study examined the emotional and psychological problems related to workplace loneliness from the perspectives of employees in the hotel service industry. It also investigated the correlation between negative emotions and potential mechanisms.

## 2. Literature Review and Conceptual Model 

### 2.1. Definition and Previous Research on Workplace Loneliness

The condition of general loneliness has been studied for a long time, but limited research has been conducted on workplace loneliness. According to Weiss [1], loneliness can take two forms: emotional isolation from a lack of friendly relationships, and social isolation from the absence of interpersonal connections. Prinz [23] classified loneliness as an emotion, whereas Barrett et al. [24] defined it as a sensory process. Nevertheless, these definitions do not entirely clarify what loneliness is. Inherently speaking, loneliness is an individual experience [25] that can differ depending on context, environment, and situation. Wright et al. [26] defined workplace loneliness as “the negative reflection in the quality of individual relations and social interactions with employees in working.” The authors demonstrated that this condition is a two-dimensional structure; that is, it is formed by emotional deprivation and the lack of social companionship at work. Yilmaz [27] referred to workplace loneliness as “solitude stemming and isolation from the social environment in workplace,” while Ozcelik and Barsade [9] defined it as the psychological pain of relationship scarcity in work settings. The causes of loneliness among employees are their inability to socialize and their inexperience with respect to the quality of interpersonal relationships. As asserted by Erdil and Ertosun [28], “workplace-specific emotion and coexists with certain character of the working environment such as competitive working climate and alternative working arrangements”.

### 2.2. Hypothesis Development

Loneliness is an unpleasant psychological emotion that can be associated with mental-health problems. For instance, workplace loneliness significantly exacerbates the stress that employees feel in their jobs, and employees who do not experience loneliness more effectively handle work-related stress [29]. This observation is supported by Roe et al. [30], who stated that low levels of loneliness are related to high levels of recovery. The authors added that recovery from job stress can be an important accomplishment for individuals and can generally be achieved through diversionary strategies and psychological detachment. Psychological detachment improves mental health and thereby stimulates pleasure in work, as indicated by Sonnentag and Bayer [20]. Additionally, Shimazu et al. [31] found a positive relationship between psychological detachment and mental health. In this study, workplace loneliness was defined as a two-dimensional construct constituted by the emotional and social domains [26]. Wright [29] said that employees who feel loneliness at work intrinsically want to get out of their environment psychologically through emotional and social detachment, and that workplace loneliness and psychological detachment have a positive relationship. In addition, Firoz and Chaudhary [32] found that employees who feel lonely in the organization for various reasons distance themselves socially and emotionally from their colleagues at work. Wright et al. [26] stated that emotional deprivation and psychological separation can be experienced through organizational loneliness. On the basis of the discussion above, the following hypotheses were formulated:

**Hypothesis** **1** **(H1).** *Workplace loneliness is positively related to psychological detachment.*

**Hypothesis** **1a** **(H1a).** *Emotional deprivation is positively related to psychological detachment.*

**Hypothesis** **1b** **(H1b).** *The lack of social companionship is positively related to psychological detachment.*

Hobfoll [33] suggested that employees suffer from burnout when their resources are inappropriately replenished. When they are unable to achieve psychological detachment, their already-reduced work resources become insufficient [34]. According to Sonnentag et al. [35], energy exhaustion may result from tiredness caused by emotions over time, especially in the absence of psychological detachment during off hours—a situation that renders the supplementation of compensatory resources difficult. Low psychological detachment also aggravates emotional exhaustion and increases the time for recovery needed by employees [36]. Derks and Bakker [37] and Derks et al. [38] demonstrated that smartphone use for work obstructs psychological detachment and that this obstruction increases exhaustion from work. Kilroy et al. [21] found that under considerable psychological detachment, emotional exhaustion decreases. As previously stated, Muhamad et al. [22] asserted that high levels of psychological disconnection increase the negative relationship between burnout and emotional resources. In line with the reasoning in this paragraph, the current work developed the following supposition:

**Hypothesis** **2** **(H2).** *Psychological detachment is negatively related to emotional exhaustion.*

Peplau and Perlman [4] defined loneliness as an undesirable feeling induced by discrepancies between perceived and desired interpersonal relationships. Loneliness also reflects discontent with existing emotional and social connections with other people [39] and is associated with a number of disadvantages, including weak social relationships and emotional health problems [40]. Occasionally, however, loneliness may not be the outcome of social isolation but is linked to the emotional exhaustion caused by burnout at work [41]. Emotional exhaustion is the core dimension of employee burnout [42], encompassing weariness, the scarcity of energy, and the lack of emotional resources [43]. Lin and Huang [44] found that college students suffering from severe loneliness exhibit many symptoms of learning-related burnout. Similarly, medical employees who experience loneliness exhibited high level suffer from severe burnout [12], and managers working in small- to medium-sized businesses are at a high risk of developing the condition [45]. Furthermore, Anand and Mishra [13] found a positive relationship between emotional exhaustion and workplace loneliness in the nursing context. Finally, George et al. [46] uncovered nonsignificant correlations among burnout, age, tenure of experience, and loneliness. In accordance with the results discussed above, the present research put forward Hypothesis 3:

**Hypothesis** **3** **(H3).** *Workplace loneliness is positively related to emotional exhaustion.*

**Hypothesis** **3a** **(H3a).** *Emotional deprivation is positively related to emotional exhaustion.*

**Hypothesis** **3b** **(H3b).** *The lack of social companionship is positively related to emotional exhaustion.*

### 2.3. Research Model

**Figure 1:**
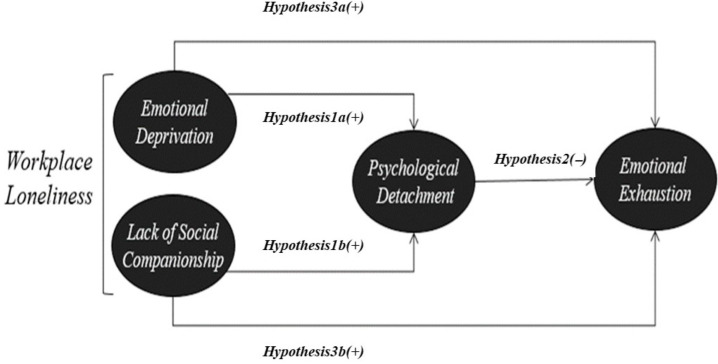
The proposed model of workplace loneliness, psychological detachment, and emotional exhaustion.

As shown in Figure 1, emotional deprivation and the lack of social companionship in the workplace were treated as the independent variables, psychological detachment served as the moderating variable, and emotional exhaustion was used as the dependent variable. To reiterate, this study investigated the effects of workplace loneliness on psychological detachment (*Hypotheses 1*), the effects of psychological detachment on emotional exhaustion (*Hypothesis 2*), and the effects of emotional deprivation and the lack of social companionship in the workplace on emotional exhaustion (*Hypothesis 3*). 

## 3. Research Methodology

### 3.1. Sampling and Data Collection

From August to September 2019, data were collected from employees of deluxe hotels in Seoul, Korea. The targeted sample was composed specifically of employees working in deluxe hotels. The employees were informed that their participation was voluntary and that their responses to a self-administered questionnaire would be anonymized. Upon approval by the human resources managers of the establishments, the employees were asked to fill in the questionnaires. A pilot test involving 40 hotel employees was conducted to determine the reliability of the scales, after which the questionnaire was revised several times on the basis of feedback from the respondents. The main survey involved 350 employees, among whom 338 returned their questionnaires. The elimination of incomplete questionnaires left a final sample of 300 employees, corresponding to an effective response rate of 85.71%. Among the participants, 68.7% were male and 31.3% were female; 37.3% were 30 to 39 years old, whereas 46.3% were 20 to 29 years old; 64% worked in the culinary department; and 79.3% were permanent employees. Most of the employees had community college degrees (44.7%) or university degrees (41.3%). Of the participants, 62.6% had been working for the case hotels for over three years. 

### 3.2. Instrument Development

The questionnaire consisted of four sections, of which the first was intended to inquire into demographic (e.g., gender, age) and job-related (e.g., department, tenure) characteristics. The second section required the employees to rate the loneliness that they experience in the workplace (Table 1). To measure workplace loneliness, this study adapted the multi-item scales of Wright [29] and Wright et al. [26]. The 12 items were rated on a seven-point scale (1 = *strongly disagree*, 7 = *strongly agree*) in response to the statement “I often feel abandoned by my co-workers when I am under pressure at work.” These items were grouped under two dimensions of workplace loneliness [26]: emotional deprivation (seven items) and the lack of social companionship (five items). The third section revolved around the employees’ psychological detachment, which was measured with four items rated on a seven-point scale based on that developed by Sonnentag and Fritz [47]. The fourth section centered on the employees’ emotional exhaustion, which was measured with seven items rated on a seven-point scale grounded in that developed by Maslach and Jackson [48].

### 3.3. Data Analysis

To collect data, we used the two-step approach recommended by Anderson and Gerbing [49], and to analyze the data, we employed the Statistical Package for the Social Sciences (version 26.0) and the Analysis of Moment Structures (version 24.0). To verify the results on demographic characteristics, frequency analysis was performed. The validity and reliability of the measured items were determined through reliability analysis and confirmatory factor analysis (CFA), and the directivity between factors was verified via correlation analysis. The proposed model, the three hypotheses, and the causal relationships indicated in the hypotheses were verified using structural equation modeling (SEM).

## 4. Results

### 4.1. Descriptive Statistics

Table 1 presents the descriptive statistics of each item in relation to the constructs of interest in this study: workplace loneliness, psychological detachment, and emotional exhaustion. Using the seven-point scales, the respondents were able to denote their perceptions of these constructs (one = minimum, four = median, seven = maximum). The mean value of each item under emotional deprivation ranged from 2.75 to 3.14. The respondents ranked “I experience a general sense of emptiness when I am at work” (3.14 ± 1.54) as the highest source of emotional deprivation in the workplace loneliness, followed by “I often feel emotionally distant from the people I work with” (3.05 ± 1.46). They ranked “I often feel abandoned by my co-workers when I am under pressure at work” (2.75 ± 1.49) as the weakest cause of emotional deprivation. In terms of the lack of social companionship, the respondents ranked “I have someone at work I can spend time with on my breaks if I want to” (3.18 ± 1.39) as the most influential factor for the lack of social companionship but rated “There is someone at work I can talk to about my day to day work problems if I need to” (2.77 ± 1.18) as the least influential. The participants scored 3.07 to 3.71 on the seven-point scale for psychological detachment. In detail, they indicated the statement “During my nonwork time I distance myself from my work” (3.71 ± 1.59) as the attribute most contributory to psychological detachment and the statement “During my nonwork time I get a break from the demands of work” (3.07 ± 1.53) as the least contributory. As for emotional exhaustion, the statement “I feel used up at the end of the workday” (4.64 ± 1.41) received the highest rating among the four attributes.

### 4.2. Measurement Model

As mentioned previously, this study followed the two-step approach suggested by Anderson and Gerbing [49]. First, CFA was carried out to evaluate the overall fit of the three-factor model consisting of workplace loneliness, psychological detachment, and emotional exhaustion. Table 2 shows the results of the CFA regarding the theory being advanced through the three-factor model. All standardized estimates exceeded 0.70, and each indicator t-value was no less than 8.00 (*p* < 0.001) [49]. The CCR ranged from 0.86 to 0.94, the Cronbach’s alpha ranged from 0.85 to 0.94, and each measurement scale was no less than the minimum requirement of 0.70. These values mean that the results reflected unidimensionality and internal consistency with respect to the relevant constructs [50]. In addition, the average variance extracted (AVE) estimates (emotional deprivation = 0.72, lack of social companionship = 0.56, psychological detachment = 0.69, and emotional exhaustion = 0.62) exceeded 0.50, indicating acceptability [51].

Discriminant validity was evaluated by comparing the squared correlation between the constructs using the AVE (See Table 3). Discriminant validity was evidenced by all the squared correlations ranging from 0 to 0.32 for each pair of constructs; the values did not exceed the AVE estimates, which ranged from 0.56 to 0.72. These results showed that the seven factors were separate and unidimensional. The confirmatory measurement models also verified the soundness of the measurement properties (χ^2^ = 728.94 (df = 224), *p* < 0.001, χ^2^/df = 3.25, goodness of fit index (GFI) = 0.81, normed fit index (NFI) = 0.87, Tucker–Lewis Index (TLI) = 0.91, comparative fit index (CFI) = 0.91, incremental fit index (IFI) = 0.91, root mean square error of approximation (RMSEA) = 0.08). 

### 4.3. Structural Equation Modeling

The proposed model and relationships (hypotheses) were tested via SEM. Table 4 shows the estimated model, standardized path coefficients, and connected t-values for all the relationships of interest [52,53]. SEM was carried out to test Hypotheses 1 to 3; the fit of the overall model was presented in consideration of the modification indices [54,55] (χ2 = 583.450, df = 220, *p* < 0.001, χ2/df = 2.65, GFI = 0.84, IFI = 0.93, CFI = 0.93, RMSEA = 0.07). Hypothesis 1 (H1a, H1b), which suggests that when workplace loneliness increases, so does psychological detachment, was supported. Emotional deprivation increased, thus elevating psychological detachment (β = 0.17, *t* = 2.15, *p* < 0.05). Similarly, when the absence of social companionship increased, psychological detachment rose (β = 0.19, *t* = 2.41, *p* < 0.05). These findings indicate that as the employees experienced increasing loneliness in the workplace, they wanted to be psychologically disconnected from their jobs. Hypothesis 2, which maintains that when psychological detachment decreases, so does emotional exhaustion, was unsupported. According to the employees who increasingly desired detachment, they suffered from progressive emotional exhaustion (β = 0.16, *t* = 2.75, *p* < 0.01). Hypothesis 3 (H3a, H3b) was partially supported. As emotional deprivation increased, so did emotional exhaustion (β = 0.43, *t* = 5.38, *p* < 0.001), but contrary to our expectations, when the lack of social companionship increased, emotional exhaustion did not (β = 0.06, *t* = 0.82, *p* > 0.05). Additionally, as a result of investigating the indirect effects of emotional deprivation (β = 0.02, *p* > 0.05) and lack of social companionship (β = 0.03, *p* > 0.05) on emotion exhaustion by mediating psychological detachment, no significant indirect effects were found.

## 5. Discussion and Implications

### 5.1. Discussion of Results

Through studies of loneliness, we know that this condition is a painful, unpleasant experience and that such an undesirable emotion extends to the workplace. The present study was aimed at establishing whether workplace loneliness in the hotel industry affects psychological detachment and emotional exhaustion among employees. The results showed evidence of workplace loneliness among the participating hotel employees. When the respondents felt lonely at work, they wanted to psychologically detach themselves from their jobs to recover. It can be seen that employees try to overcome their unpleasant feelings by staying away from their job or company during their nonwork time. The study also uncovered that workplace loneliness contributed to emotional exhaustion, which partially aligns with the results of earlier studies [12,13]. Through these results, it was found that there was a significant correlation between employees’ psychological and emotional problem. In addition, emotional factors had more influence on emotional exhaustion than social factors, and psychological detachment did not entirely eliminate the emotional exhaustion felt by the employees.

### 5.2. Theoretical and Practical Implications

Few empirical studies have been devoted to the effects of loneliness in the workplace on the psychological detachment and emotional exhaustion of employees in the hotel service industry. Compared with employees in other industries, hotel employees work with near-reciprocal social actions because of the nature of their work conditions, but minimal studies have been performed on these individuals because employees’ emotional and psychological problems are sensitive subjects. Nevertheless, empirical research is necessary to determine whether workplace loneliness significantly affects psychological detachment and emotional exhaustion among employees. The current work therefore contributes to research on these matters in the hotel service industry and to the comprehension of the psychological problems encountered by employees. It also offers a theoretical, academic foundation from which to explore workplace loneliness, which may motivate further research, seeing as extant scholarship focused on comparing simple causal relationships with respect to the psychological and emotional problems experienced by employees. 

The present study presents two significant practical implications. First, the results suggest that psychologically detaching from work is important when employees experience workplace loneliness. Wright [29] indicated that workplace loneliness, which encompasses negative mental health-related outcomes, can be addressed through psychological detachment, which leads to positive outcomes at work [20]. Furthermore, previous studies identified a host of advantageous effects from psychological detachment, such as decreased exhaustion and increased positivity in atmosphere [35,56]. Correspondingly, employees should be educated about the benefits of psychological detachment at the organizational level, and managers should play a more assertive role in ensuring that their subordinates have acceptable opportunities for psychological disconnection from work. To this end, practical policies such as support for leisure activities and psychological programs for members of the organization should be prepared. It will also be necessary to establish a mentoring system to establish an informal interview system with a supervisor whom they trust and follow. Second, this study found that workplace loneliness can closely predict emotional exhaustion among employees; that is, exhaustion is the result of loneliness in the workplace. Cordes and Dougherty [57] suggested that exhaustion is associated with significant costs for both individuals and organizations. Managers should therefore consider involvement to reduce emotional exhaustion by minimizing experiences of loneliness in the work setting. Moreover, Wright et al. [26] explained workplace loneliness as reflecting discontent with the quality of connections among employees. The hotel service industry—where work entails social interaction between employees—should ensure that the work atmosphere is conducive to relationships and companionship at the organizational level. Considering loneliness as part of normal life, individuals may experience varying levels of this condition, but severe occurrence in the workplace should be treated as an important risk factor. Broadly speaking, conducting increased explorations of social relationships and environments at work may be beneficial in understanding the roles of organizational behaviors and improving both personal and organizational connections. Given the complicated nature of personal social environments, companionship between co-workers, loneliness, and organizations, this study has contributed to the understanding of the possibility that employees feel lonely in the workplace. Additionally, workplace loneliness can serve as an opportunity to recognize that loneliness is an inevitable and natural emotion that employees can experience in their organizational life. It provided important implications for organizations to effectively manage loneliness. 

### 5.3. Limitations and Future Research

First, because this research involved only employees of hotels in Seoul, Korea, and because they were asked to fill in self-administered questionnaires, it is difficult to definitively determine generalizability. Further research should be conducted to include various employees from other industries. Second, this study did not measure workplace loneliness on the basis of local culture. To address this issue, researchers should develop a scale in a way that corresponds with the collectivist culture of Koreans. This approach can also overcome problems caused by translation into the Korean mother tongue. Finally, even though the labor-intensive structure of the hotel industry paves the way for the identification of numerous correlations, research that extends to other sectors and industries can increase the reliability and validity of the study. Investigations of workplace loneliness are concerned with the assessment of personal social environments, rather than merely an individual’s characteristics in themselves. Future research would therefore benefit from scrutinizing how an individual’s personality influences how they correlate with and analyze their social environments in the workplace.

## Figures and Tables

**Table 1:** Descriptive statistics of variables.

Items	Mean ± SD
Workplace Loneliness: Emotional Deprivation	
ED_1_ I often feel abandoned by my co-workers when I am under pressure at work	2.75 ± 1.49
ED_2_ I often feel alienated from my co-workers	2.80 ± 1.49
ED_3_ I feel myself with drawing from the people I work with	2.75 ± 1.49
ED_4_ I often feel emotionally distant from the people I work with	3.05 ± 1.46
ED_5_ I often feel isolated when I am with my co-workers	2.83 ± 1.42
ED_6_ I often feel disconnected from others at work	2.89 ± 1.47
ED_7_ I experience a general sense of emptiness when I am at work	3.14 ± 1.54
Workplace Loneliness: Lack of Social Companionship	
SC_1_ I have social companionship/fellowship at work	3.05 ± 1.21
SC_2_ There is someone at work I can talk to about my day to day work problems if I need to	2.77 ± 1.18
SC_3_ I have someone at work I can spend time with on my breaks if I want to	3.18 ± 1.39
SC_4_ I feel part of a group of friends at work	2.99 ± 1.18
SC_5_ There are people at work who take the trouble to listen to me	3.05 ± 1.24
Psychological Detachment	
PD_1_ During my nonwork time I forget about work	3.57 ± 1.60
PD_2_ During my nonwork time I don’t think about work at all	3.42 ± 1.55
PD_3_ During my nonwork time I distance myself from my work	3.71 ± 1.59
PD_4_ During my nonwork time I get a break from the demands of work	3.07 ± 1.53
Emotional Exhaustion	
EE_1_ I feel used up at the end of the workday	4.64 ± 1.41
EE_2_ I feel fatigued when I get up in the morning and have to begin a day	3.96 ± 1.44
EE_3_ Working with people all day is really a strain for me	3.78 ± 1.46
EE_4_ I feel burned out from my job	4.01 ± 1.45
EE_5_ I feel frustrated by my job	3.87 ± 1.54
EE_6_ I feel I’m working too hard on my job	3.78 ± 1.49
EE_7_ Working with people directly puts too much stress on me	3.34 ± 1.50

Note: (1) SD—standard deviation; (2) ED—emotional deprivation; SC—lack of social companionship; PD—psychological detachment; EE—emotional exhaustion.

**Table 2:** Correlation analysis.

Construct	1	2	3	4	5	6	7	8
1. Gender	1							
2. Age	−0.21 **	1						
3. Education level	0.02	0.19 **	1					
4. Tenure	−0.13 *	0.81 **	0.23 **	1				
5. ED	0.11	0.19 **	0.06	0.15 *	1			
6. SC	0.00	0.00	0.01	0.00	0.57 **	1		
7. PD	0.17 **	0.01	−0.04	0.13 *	0.27 **	0.27 **	1	
8. EE	0.12 *	0.01	0.06	0.05	0.47 **	0.31 **	0.28 **	1

Note: (1) All variables were measured on a 7-point Likert scale from 1 (strongly disagree) to 7 (strongly agree); ** *p* < 0.01, * *p* < 0.05; (2) ED—emotional deprivation; SC—lack of social companionship; PD—psychological detachment; EE—emotional exhaustion.

**Table 3:** Characteristics of reliability and confirmatory factor analyses.

Construct	Standardized Estimate	*t*-Value	CCR ^a^ (AVE ^b^)	Cronbach’s Alpha
Emotional Deprivation			0.94	0.94
			(0.72)	
ED_1_	0.77	fixed		
ED_2_	0.86	16.72 ***		
ED_3_	0.86	16.96 ***		
ED_4_	0.89	17.59 ***		
ED_5_	0.91	18.06 ***		
ED_6_	0.87	16.97 ***		
ED_7_	0.76	14.38 ***		
Lack of Social Companionship			0.86	0.85
			(0.56)	
SC_1_	0.71	fixed		
SC_2_	0.62	18.17 ***		
SC_3_	0.61	11.18 ***		
SC_4_	0.89	11.46 ***		
SC_5_	0.84	13.76 ***		
Psychological Detachment			0.89	0.89
			(0.69)	
PD_1_	0.88	fixed		
PD_2_	0.91	22.21 ***		
PD_3_	0.85	19.83 ***		
PD_4_	0.64	12.66 ***		
Emotional Exhaustion			0.91	0.92
			(0.62)	
EE_1_	0.58	fixed		
EE_2_	0.83	10.79 ***		
EE_3_	0.81	10.67 ***		
EE_4_	0.85	10.92 ***		
EE_5_	0.83	10.82 ***		
EE_6_	0.86	11.02 ***		
EE_7_	0.70	9.66 ***		

Note: ^a^ CCR—composite construct reliability; ^b^ average variance extracted; χ^2^ = 728.94 (df = 224) *p* < 0.001; χ^2^/df = 3.25; goodness of fit index (GFI) = 0.81; normed fit index (NFI) = 0.87; Tucker–Lewis index (TLI) = 0.91; comparative fit index (CFI) = 0.91; Incremental Fit Index (IFI) = 0.91; root mean square error of approximation (RMSEA) = 0.08; *** *p* < 0.001.

**Table 4:** Structural parameter estimates.

Stated as Alternative Hypothesis	Standardized Path Coefficients	S.E.	*t*-Value	Results
H1a Emotional Deprivation →Psychological Detachment	0.17	0.09	2.15 *	Supported
H1b Lack of Social Companionship →Psychological Detachment	0.19	0.10	2.41 *	Supported
H2 Psychological Detachment →Emotional Exhaustion	0.16	0.03	2.75 **	Supported
H3a Emotional Deprivation →Emotional Exhaustion	0.43	0.05	5.38 ***	Supported
H3b Lack of Social Companionship →Emotional Exhaustion	0.06	0.05	0.82	Not Supported
χ^2^	583.450			
df	220			
χ^2^/df	2.65			
GFI	0.84			
IFI	0.93			
CFI	0.93			
RMSEA	0.07			

Note: GFI—goodness of fit index; IFI—incremental fit index; CFI—comparative fit index; RMSEA—root mean square error of approximation; * *p* < 0.05, ** *p* < 0.01, *** *p* < 0.001.

## Data Availability

The data presented in this study are available on request from the first author.
